# Incorporation of mineral nitrogen into the soil food web as affected by plant community composition

**DOI:** 10.1002/ece3.7325

**Published:** 2021-03-25

**Authors:** Tanja Strecker, Annette Jesch, Dörte Bachmann, Melissa Jüds, Kevin Karbstein, Janneke Ravenek, Christiane Roscher, Alexandra Weigelt, Nico Eisenhauer, Stefan Scheu

**Affiliations:** ^1^ J.F. Blumenbach Institute of Zoology and Anthropology University of Göttingen Göttingen Germany; ^2^ German Centre for Integrative Biodiversity Research Halle‐Jena‐Leipzig Leipzig Germany; ^3^ Institute of Agricultural Sciences ETH Zurich Zürich Switzerland; ^4^ Department of Systematics, Biodiversity and Evolution of Plants University of Göttingen Göttingen Germany; ^5^ Department of Experimental Plant Ecology Institute for Water and Wetland Research Radboud University Nijmegen The Netherlands; ^6^ Institute of Biology Leipzig University Leipzig Germany; ^7^ Department of Physiological Diversity Helmholtz Centre for Environmental Research, UFZ Leipzig Germany; ^8^ Department of Systematic Botany and Functional Biodiversity, Institute of Biology Leipzig University Leipzig Germany; ^9^ Centre of Biodiversity and Sustainable Land Use University of Göttingen Göttingen Germany

**Keywords:** food, grassland, microarthropods, nutrient channeling, soil fauna

## Abstract

Although nitrogen (N) deposition is increasing globally, N availability still limits many organisms, such as microorganisms and mesofauna. However, little is known to which extent soil organisms rely on mineral‐derived N and whether plant community composition modifies its incorporation into soil food webs. More diverse plant communities more effectively compete with microorganisms for mineral N likely reducing the incorporation of mineral‐derived N into soil food webs. We set up a field experiment in experimental grasslands with different levels of plant species and functional group richness. We labeled soil with ^15^NH_4_^15^NO_3_ and analyzed the incorporation of mineral‐derived ^15^N into soil microorganisms and mesofauna over 3 months. Mineral‐derived N incorporation decreased over time in all investigated organisms. Plant species richness and presence of legumes reduced the uptake of mineral‐derived N into microorganisms. In parallel, the incorporation of mineral‐derived ^15^N into mesofauna species declined with time and decreased with increasing plant species richness in the secondary decomposer springtail *Ceratophysella* sp. Effects of both plant species richness and functional group richness on other mesofauna species varied with time. The presence of grasses increased the ^15^N incorporation into *Ceratophysella* sp., but decreased it in the primary decomposer oribatid mite *Tectocepheus velatus sarekensis*. The results highlight that mineral N is quickly channeled into soil animal food webs via microorganisms irrespective of plant diversity. The amount of mineral‐derived N incorporated into soil animals, and the plant community properties affecting this incorporation, differed markedly between soil animal taxa, reflecting species‐specific use of food resources. Our results highlight that plant diversity and community composition alter the competition for N in soil and change the transfer of N across trophic levels in soil food webs, potentially leading to changes in soil animal population dynamics and community composition. Sustaining high plant diversity may buffer detrimental effects of elevated N deposition on soil biota.

## Graphical abstract

Soil mineral nitrogen is incorporated quickly into microorganisms and higher trophic levels of the soil food web. Incorporation is not affected by plant species richness, but by plant community composition with legumes diluting the incorporation of soil mineral nitrogen. This highlights that plant community composition alters the competition for nitrogen in soil and changes nitrogen transfer across trophic levels in soil food webs, potentially leading to changes in soil animal population dynamics and community composition.

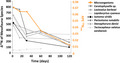

## 1 INTRODUCTION

Soil microorganisms and soil fauna are key players for ecosystem functions such as decomposition and element cycling. Nitrogen (N) mineralization is an almost entirely microbially driven process (Veresoglou et al., 2012), but soil animals also contribute directly or indirectly to N cycling (Carrillo et al., 2011; Seastedt, 1984; Verhoef & Brussaard, 1990). Soil animal species can be classified into primary decomposers, secondary decomposers, and predators (Scheu, 2002). Primary decomposers contribute to decomposition and mineralization of nutrients by feeding on dead plant material, while secondary decomposers mainly feed on microorganisms living in soil or being associated with plant roots. Both primary and secondary decomposers, such as Oribatida and Collembola, significantly contribute to carbon (C) and N cycling (Filser, 2002; Lemanski & Scheu, 2015; Osler & Sommerkorn, 2007; Pollierer et al., 2012; Verhoef & Brussaard, 1990). The beneficial effects of these animal groups on C and N cycling are mostly indirect either via modifying microbial activity (Buscot & Varma, 2005) or via distributing microbial propagules (Filser, 2002; Renker et al., 2005). However, the sources from which soil animals acquire N for their own nutrition and the factors affecting N acquisition by soil animals are little understood.

Nitrogen is an essential, but limiting resource for plants and soil animals (Sterner & Elser, 2003; Vitousek & Howarth, 1991; White, 1993). Plants and saprotrophic microorganisms predominantly take up mineral N and incorporate it into tissue compounds, in particular proteins, the predominant source of N for animal nutrition. Despite the central role of N for soil animal nutrition, most of the studies that investigated element fluxes in soil food webs focused on the flux of C (Albers et al., 2006; Müller et al., 2016; Pollierer et al., 2007), and little is known about the resources soil animals use to meet their N requirements. Generally, soil animals, such as primary and secondary decomposers, meet their demand for N based either on microbial N or on plant litter N. However, the relative contribution of these sources for soil animal N nutrition is little understood. Pollierer et al. (2012) demonstrated that soil animals in beech forest use both the microbial energy channel (bacteria and fungi) and plants as C sources. Thus, soil animals may satisfy their demand for N also by both channels. Zieger et al. (2017) and Scheunemann et al. (2016) showed that decomposer mesofauna species in beech forests and arable fields gain C as well as N by feeding on microorganisms, especially fungi.

The acquisition of N by soil animals for building up their body tissue likely also varies with environmental factors influencing soil animal nutrition. In particular, plants may modify the incorporation of N by soil animals as they compete with microorganisms for N in soil and thereby alter the availability of microbial N for soil animals (Kuzyakov & Xu, 2013; Strecker et al., 2015). Simultaneously, plants may provide soil microorganisms and animals with C and N via rhizodeposition (Schenck zu Schweinsberg‐Mickan et al., 2012; Zieger, Holczinger, et al., 2017). Recent studies showed that plant diversity increases soil N storage (Oelmann et al., 2011), soil microbial activity and C storage (Lange et al., 2015), and soil microbial biomass (Eisenhauer et al., 2010, 2017; Strecker et al., 2015). Scherber et al. (2010) demonstrated cascading effects of plant diversity on the whole animal food web in temperate experimental grasslands, which might be due to plant‐mediated changes in animal N nutrition. For understanding the impact of plant diversity on the soil animal food web, the role of plants for the nutrition of soil animals needs closer consideration (Chahartaghi et al., 2005; Crotty et al., 2011; Sechi et al., 2014).

Plants compete with soil microorganisms for mineral N as both typically are limited by N (Hodge et al., 2000; Kuzyakov & Xu, 2013), and this likely is more severe in species‐rich plant communities as plants take up nutrients more efficiently in more diverse communities (Bessler et al., 2012; Jesch et al., 2018; Roscher et al., 2008). Eisenhauer et al. (2013) investigated the effects of plant diversity and N deposition on the abundance and diversity of soil fauna, but did not consider the incorporation of N into soil animals. With the present study, we addressed these gaps by investigating the incorporation of mineral N into soil microorganisms and subsequently into soil mesofauna species as modified by plant diversity.

Besides plant diversity, plant functional groups, such as grasses and legumes, may affect nutrient incorporation into soil mesofauna as they have different root C‐to‐N ratios (Chen et al., 2008), and differ in their annual N uptake (Bessler et al., 2012). Grasses are highly competitive for soil N due to their dense root system and clonal growth (Hodge et al., 1999; Roscher et al., 2008; de Witte & Stöcklin, 2010). Strecker et al. (2015) observed the microbial C‐to‐N ratio to increase in the presence of grasses at the field site of the present study, indicating competition for N between microorganisms and grasses. This finding is supported by the results of Oelmann et al. (2007) who found reduced soil mineral N concentration in the presence of grasses at the same field site. Consequently, soil animal species relying on microbial N likely also experience increased N limitation in the presence of grasses. Legumes can fix molecular N via rhizobia and fuel the soil system with organic N via rhizodeposition and input of litter material. Thereby, legumes are likely to mitigate competition for N and influence the N nutrition of soil microorganisms and soil animals (Marschner et al., 2011; Milcu et al., 2006; Oelmann et al., 2007; Spehn et al., 2002).

In the present study, we aimed at tracking the incorporation of labeled mineral N into soil microorganisms and the channeling of the incorporated mineral N to higher trophic levels of the soil food web as affected by plant diversity and plant community composition in experimental temperate grassland. ^15^N stable isotope labeling was used for tracing N fluxes into different compartments of the belowground system (Crotty et al., 2012; Zieger et al., 2015). As the channeling of N from lower to higher trophic levels likely occurs with a time lag, we expected the ^15^N signal to be incorporated first into soil microorganisms, then into secondary decomposers, and finally into predatory species, but not into primary decomposers (using only plant litter as food). To test these expectations, we followed the incorporation of ^15^N into soil microorganisms and mesofauna 2, 15, 30, 60, and 120 days after labeling.

In detail, we investigated the following hypotheses:

1. Incorporation of mineral N into mesofauna taxa generally follows that of the incorporation of mineral N into soil microorganisms, but this is less pronounced in primary decomposers and predators than in secondary decomposers.
2. Incorporation of mineral N into soil microorganisms and mesofauna decreases with increasing plant diversity (plant species richness and functional group richness), as high plant diversity communities more efficiently exploit nutrient resources in soil.
3. Presence of legumes decreases the incorporation of mineral N into microorganisms and mesofauna, as legumes fuel the soil system with biologically fixed N.
4. Presence of grasses decreases the incorporation of mineral N into microorganisms and mesofauna, as grasses effectively compete for soil N with other soil organisms.

## 2 MATERIALS AND METHODS

### 2.1 Study site

The experiment was conducted within the framework of the Jena Experiment, a large grassland biodiversity experiment, which investigates in an integrative way the role of plant diversity for ecosystem functioning (Roscher et al., 2004). The experiment was established in 2002 on a former arable fields in the floodplain of the Saale River near to the city of Jena (Thuringia, Germany; 50°55′N, 11°35′E, 130 m a.s.l.). The soil is Eutric Fluvisol, mean precipitation is 610 mm per year, and mean temperature is 9.9°C (Hoffmann et al., 2014). The plant species used in the experiment are typical for Central European mesophilic grasslands (Arrhenatherion community; Ellenberg & Leuschner, 2010). The experimental plots did not receive any fertilizer and were mown twice a year, and aboveground plant biomass was removed from the field site to imitate typical management of extensive hay meadows in the study region and weeded by hand two to three times a year to maintain the target plant community composition.

The 80 plant communities were selected out of a pool of 60 plant species and comprised a plant species richness gradient including monocultures and 2, 4, 8, 16, and 60 plant species combinations. The plant species were chosen from four plant functional groups and selected according to cluster analyses based on above‐ and belowground morphological traits, phenological traits, and N_2_ fixation, resulting in 16 grasses, 12 small herbs, 20 tall herbs, and 12 legumes. Thus, the plant communities also comprised a plant functional group richness gradient (1, 2, 3, and 4 plant functional groups). The plots were grouped into four blocks with an equal number of plots per diversity level to account for changes in soil texture with increasing distance from the Saale River. For detailed information on the design of the Jena Experiment see Roscher et al. (2004).

### 2.2 Experimental design

In this study, a subset of 40 plots varying in plant species richness (2, 4, 8, and 16 plant species) was used, with ten replicates per plant species richness level. The number of plant functional group richness levels (1, 2, 3, and 4 plant functional groups) was balanced within each species richness level, and the plots with different diversity levels were equally distributed across the four blocks. For more details on the selection of plant species see Roscher et al. (2004).

On each of the 40 experimental plots, a subplot (56 × 69 cm) was established from which samples were taken in five sequential sampling campaigns. To prevent horizontal flow of the tracer solution out of the subplots and to reduce lateral migration of soil animals between labeled and unlabeled areas, PVC boards were installed as barriers along the subplot border to a height and depth of 15 cm, respectively.

### 2.3 Experimental procedure

The experimental subplots were labeled with ^15^N at the beginning of the growing season in 2011 (18–19 April). The ^15^N tracer solution (0.01 mol ^15^NH_4_^15^NO_3_/L deionized water; 98 atom %; Cambridge Isotope Laboratories) was injected into predrilled holes of a depth of 7 cm in the soil arranged along gridlines (distance within grid lines 8.7 cm, distance between grid lines 10 cm, resulting in 49 holes per subplot). The tracer solution was injected using a 3 mm‐thick four‐side port needle (2 ml per injection point) connected with a silicon tube to a bottle top dispenser (Socorex Isba SA) on a 1 L glass bottle. A funnel was used to prevent contamination of the vegetation with tracer solution.

For measuring the time‐integrated incorporation of ^15^N into soil microorganisms and mesofauna, five samples were taken 2 (5 for mesofauna), 15, 30, 60, and 120 days after labeling. At each sampling campaign, three soil cores were taken per subplot for microbial biomass (Ø 5 cm, 0–5 cm depth) and one soil core for mesofauna (Ø 20 cm, 0–10 cm depth). The three samples per subplot for measuring microbial biomass were pooled, placed into plastic bags, and stored at 4°C until further analyses. Soil cores for analyzing mesofauna were stored for a maximum of 4 days at 4°C to prevent soil animals from deceasing. Soil animals were extracted with a high gradient heat extractor, collected in glycerol, transferred into 70% ethanol, and identified to species or genus level. For the analysis of the natural abundance of ^15^N, reference soil cores for microorganisms and mesofauna were taken 10 cm adjacent to the sampling area within each plot 5 days before labeling as described above.

Based on biomass estimates, the following mesofauna species were used for stable isotope analyses: *Tectocepheus velatus sarekensis* (Oribatida, primary decomposer), *Lepidocyrtus cyaneus*, *Isotoma viridis, Parisotoma notabilis, Ceratophysella* sp., and *Stenaphorura denisi* (all Collembola, secondary decomposers), and *Lasioseius berlesei* (Gamasina, predator). All of these species are abundant and widespread in grasslands and other ecosystems in Central Europe including the field site of the Jena Experiment (González‐Macé & Scheu, 2018; Sabais et al., 2011).

Microbial biomass N was extracted from soil by chloroform fumigation extraction (CFE) (Brookes et al., 1985). Prior to the extraction, roots were removed by hand. To remove other background N, 50 g fresh soil of each sample was taken, and N was removed via pre‐extraction with 100 ml 0.05 M K_2_SO_4_ with agitation for 30 min (200 rpm) and centrifugation for 10 min (200 U/min) at 4,000 *g*. Two subsamples (10 g soil fresh weight each) were taken from each pre‐extracted soil sample. One subsample was fumigated with chloroform vapor for 24 hr, and the other remained unfumigated. Both subsamples were extracted with 60 ml 0.05 M K_2_SO_4_ as described above, and the extracts were filtered and frozen at −18°C until further analysis. At each fumigation campaign, two blank samples were processed together with fumigated and unfumigated subsamples to account for contamination of the subsamples during the procedure. Before analyzing stable isotope ratios of the subsamples and blank samples, a fraction of the samples (15 ml) was freeze‐dried (VaCo2; Zirbus Technology) at −30°C for 3 days and stored in plastic vessels in a desiccator. For referring results of ^15^N measurements to one gram dry soil, gravimetric soil water content was measured by drying 10 g of fresh soil subsamples of each sample at 105°C for 48 hr.

### 2.4 Stable isotope analysis

For analyses of ^15^N/^14^N ratios in microbial biomass N and in soil mesofauna, appropriate amounts of the freeze‐dried microbial N extract (60–65 µg) and appropriate numbers of animals (10–120 individuals weighing 10–200 µg and containing 1–20 µg N) were transferred into tin capsules. In few cases, individuals from the same sampling campaign but different plots with similar plant community composition were pooled. Stable isotope ratios were measured with a coupled system of an elemental analyzer (NA 1,500; Carlo Erba) and a mass spectrometer (MAT 251; Finnigan) (Reineking et al., 1993). Mesofauna samples were measured on a micro‐elemental analyzer system (Euro‐EA 300; Eurovector) allowing the analysis of small amounts of animal tissue (Langel & Dyckmans, 2014). Isotope signatures are expressed using the δ notation with δ^15^N (‰) = (*R*_sample_/*R*_standard_ − 1) × 1000, where *R* is the molar ratio of heavy to the light isotope (^15^N/^14^N). Acetanilide (C_8_H_9_NO; Merck) was used for internal calibration. As standard for δ^15^N, atmospheric N was used. Shifts in ^15^N/^14^N ratios in mesofauna species due to labeling with ^15^NH_4_^15^NO_3_ were inspected by calculating the difference between δ^15^N values of specimens inside and outside the subplots, that is, Δ values.

For calculating microbial biomass N, the amounts of N in the two blind samples of the different CFE campaigns were averaged and subtracted from the measured N mass of each subsample. Microbial biomass N was calculated as N_mic_ = *E*_N_/*k*_EN_, with *E*_N_ being the difference between total N extracted from fumigated soil and total N extracted from unfumigated soil, and *k*_EN_ being the extractable fraction of microbial biomass N after fumigation (Joergensen & Mueller, 1996). Soil microbial biomass ^15^N (µg ^15^N/g dry soil) was calculated as ^15^N_mic_ (µg/g dry soil) = ^15^N (µg/g dry soil) of fumigated subsample – ^15^N (µg/g dry soil) of unfumigated subsample (Brookes et al., 1985). Atom percent excess (APE, isotopic enrichment) of ^15^N in microbial biomass N was calculated as the difference in atom% between labeled and natural abundance levels of ^15^N in soil microbial biomass (Buresh et al., 1982; Dyckmans et al., 2005) as APE ${}^{15}N=\left({\left(\frac{{}^{15}N_{\text{mic}}}{{\text{total N}}_{\text{mic}}}\right)}_{\text{labeled}}‐{\left(\frac{{}^{15}N_{\text{mic}}}{{\text{total N}}_{\text{mic}}}\right)}_{\text{natural}}\right)*100.$ Microbial biomass C was calculated as C_mic_ = *E*_C_/*k*_EC_, with *E*_C_ being the difference between total C extracted from fumigated soil and total C extracted from unfumigated soil.

### 2.5 Statistical analyses

All statistical analyses were performed in R vers. 3.6.1 using the graphical user interface RSTUDIO vers. 1.1.383 (R Core Team, 2019). Data were inspected for normality (Shapiro–Wilk test, Q‐Q plot) and homoscedasticity (Levene's test). To improve normality and homoscedasticity, we log‐transformed microbial APE ^15^N and Δ^15^N for the total dataset and for all animal species separately. For testing the effects of plant community properties on microbial APE ^15^N and Δ^15^N values of mesofauna species, plant species richness was log‐transformed to linearize the relationship between plant diversity and microbial properties (Hooper et al., 2005). Regarding the multiple linear regression model (LM) of the total dataset between ^15^N data and the fixed factors block (four levels), animal species (seven levels), plant species richness (SR, log‐linear term; four levels), plant functional group richness (FGR, linear term; four levels), legume presence–absence (LEG, two levels), grasses presence–absence (GR, two levels), and time (days since labeling; five levels), no experimental treatment significantly influenced the response variable except of block, animal species, and time (see Table A1). Therefore, we chose multiple linear mixed‐effects models (LMEs), used block as random factor, and calculated LMEs between ^15^N data and treatments for each animal species and microbial biomass separately. The presence–absence of grasses (GR) and the presence–absence of legumes (LEG) as well as days after labeling (time) were used as categorical predictors, while log‐transformed plant species richness (SR) and plant functional group richness (FGR) were used as linear variables (using SR and FGR as categorical predictors was not possible because the dataset was too small and unbalanced). We used the function lmer() implemented in the R package “lme4” vers. 1.1‐21 (Bates et al., 2015; Bates et al., 2019) and the R package “lmerTest” vers. 3.1‐0 (Kuznetsova et al., 2019) for calculating p‐values. We performed backward model selection by stepwise removing the least significant variable until the final model was reached (Crawley, 2015). Additionally, we proved each model simplification step with the Akaike information criterion (AIC).

## 3 RESULTS

### 3.1 Microorganisms

**FIGURE 1:**
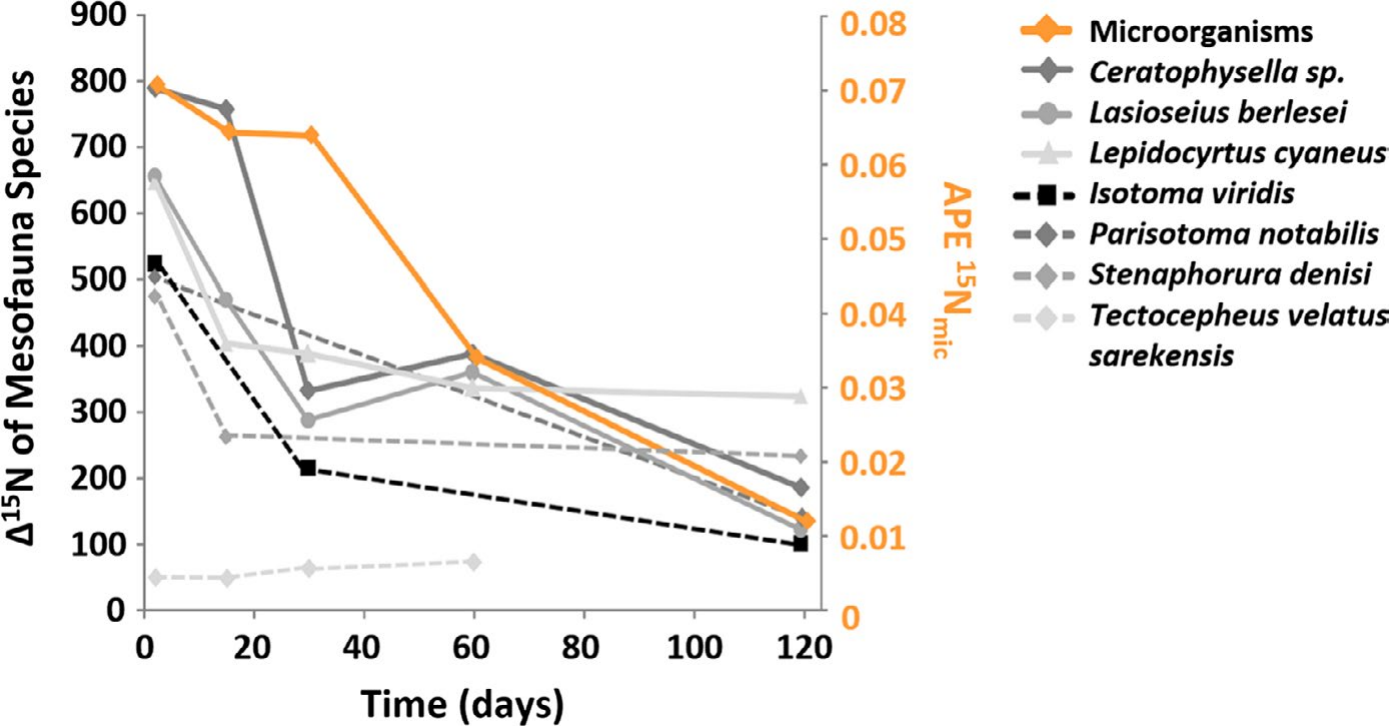
Changes in the incorporation of ^15^N into soil microorganisms (APE ^15^N_mic_) and soil mesofauna species (Δ^15^N values) over time (2–120 days for microorganisms and 5–120 days for mesofauna species)

**FIGURE 2:**
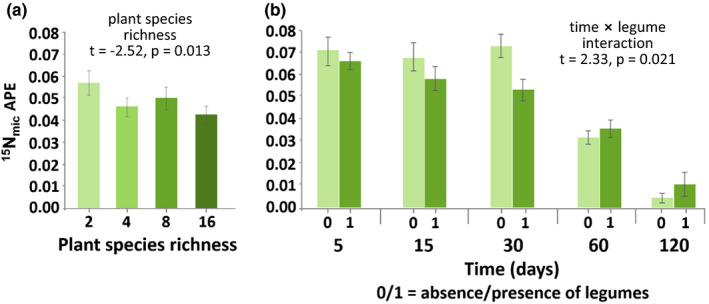
Changes in the incorporation of ^15^N into microbial biomass N (^15^N_mic_ APE) with (a) plant species richness and (b) time and presence of legumes; 0/1 = absence/presence of legumes; means ± 1 *SE*

Generally, the incorporation of ^15^N into microorganisms (^15^N_mic_ APE) declined with time (*t* = −12.78, *p* < 0.0001, Figure 1), reflecting the fast incorporation of the mineral ^15^N into microorganisms and its following turnover. Plant species richness significantly decreased ^15^N_mic_ APE (Figure 2a). The presence of legumes affected microbial ^15^N, but this effect varied over time with microbial ^15^N being lower in the presence of legumes compared to without legumes at early sampling dates, but this effect disappeared at days 60 and 120 (Figure 2b). ^15^N_mic_ (µg/g dry soil) was positively correlated with N_mic_ (*R* ^2^ = 0.93; *t* = 43.69, *p* < 0.0001) and C_mic_ (*R* ^2^ = 0.83; *t* = 26.24, *p* < 0.0001) irrespective of the sampling date, indicating that changes in ^15^N incorporation paralleled changes in N_mic_ and C_mic_.

### 3.2 Mesofauna

**FIGURE 3:**
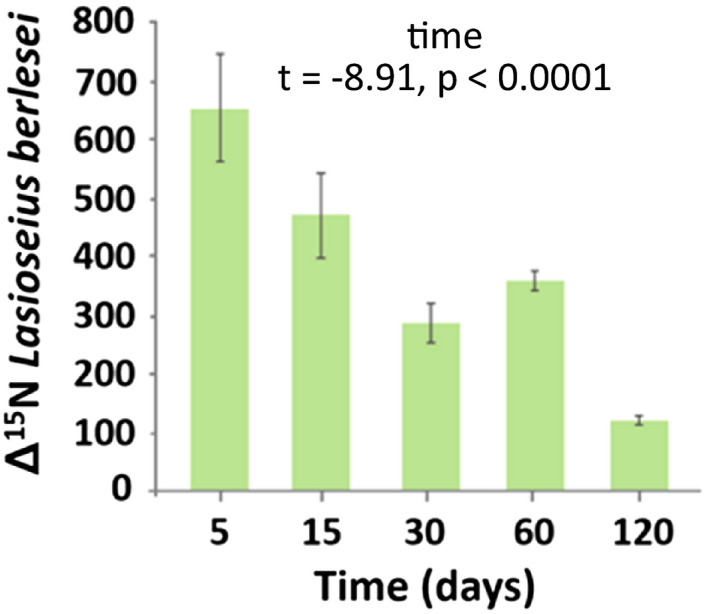
Changes in the incorporation of ^15^N into *Lasioseius berlesei* (Δ^15^N values) with time; means ± 1 *SE*

Generally, the incorporation of ^15^N (Δ^15^N values) into the studied mesofauna species declined with time parallel to ^15^N_mic_ APE, except in *T. velatus sarekensis* (Figure 1), reflecting the dominant flux of mineral N into mesofauna species via microbial N. However, the factors affecting the incorporation of ^15^N into mesofauna varied among the species studied (Table 1). Time affected the mineral ^15^N incorporation into all studied mesofauna species, either as a main factor or in combination with plant community properties. As main factor, it was only significant in *L. berlesei* and marginally significant in *L. cyaneus* with Δ^15^N values decreasing over time (Figure 3, Figure 6c, Table 1).

**TABLE 1:** Incorporation of mineral‐derived N into mesofauna species as affected by plant diversity (species richness, SR, functional group richness, FGR), plant functional group identity, (legumes, LEG, grasses, GR) and time

	*Lasioseius berlesei*				*Ceratophysella* sp.				*Tectocepheus velatus sarekensis*				*Parisotoma notabilis*				*Stenaphorura denisi*			
	Estimate	*df*	*t‐value*	*p‐value*	Estimate	*df*	*t‐value*	*p‐value*	Estimate	*df*	*t‐value*	*p‐value*	Estimate	*df*	*t‐value*	*p‐value*	Estimate	*df*	*t‐value*	*p‐value*
(Intercept)	2.711	75.00	71.27	<0.0001	2.620	10.68	24.12	<0.0001	1.792	52.00	20.69	<0.0001	2.462	16.41	22.30	<0.0001	2.449	22.00	46.25	<0.0001
SR					−0.262	64.82	−2.20	**0.032**	−0.261	52.00	−2.28	**0.026**	0.219	24.76	1.92	0.070				
FGR																				
LEG																				
GR					0.460	10.65	3.72	**0.004**												
Time	−0.005	75.00	−8.91	**<0.0001**																
SR × Time									0.007	52.00	2.25	**0.028**	−0.005	24.36	−6.67	**<0.0001**	−0.007	22.00	−2.55	**0.018**
FGR × Time									0.003	52.00	2.63	**0.011**					0.002	22.00	2.48	**0.021**
LEG × Time																	−0.005	22.00	−1.99	0.060
GR × Time					−0.004	68.99	−4.58	**<0.0001**	−0.010	52.00	−3.80	**0.0004**					0.003	22.00	1.42	0.171
AIC full mod.	−14.10				35.47				30.72				5.15				79.979			
AIC red. mod.	−25.38				31.46				−1.03				−4.84				3.35			

	*Lepidocyrtus cyaneus*				*Isotoma viridis*			
	Estimate	*df*	*t‐value*	*p‐value*	Estimate	*df*	*t‐value*	*p‐value*
(Intercept)	2.6724	8.72	61.56	<0.0001	2.36	12	11.25	<0.0001
SR					0.46	12	1.772	0.102
FGR								
LEG					−0.39	12	−1.953	0.075
GR								
Time	−0.002	63.38	−1.80	0.077				
SR × Time								
FGR × Time	−0.0009	63.51	−2.69	**0.009**	0.00	12	−2.92	**0.013**
LEG × Time					0.01	12	2.05	0.063
GR × Time								
AIC full mod.	−16.48				15.07			
AIC red. mod.	−26.57				13.20			

Note

LME table of *t*‐ and *p*‐values for the effects of the factors plant species richness (SR), plant functional group richness (FGR), presence of legumes (LEG), presence of grasses (GR), and time on the incorporation of mineral nitrogen into soil mesofauna species (Δ^15^N values). Intercept = intersection point with *y*‐axis. *df* = estimated degrees of freedom. Significant effects (*p* ≤0.05) are given in bold; *df* = theoretical degrees of freedom.

**FIGURE 4:**
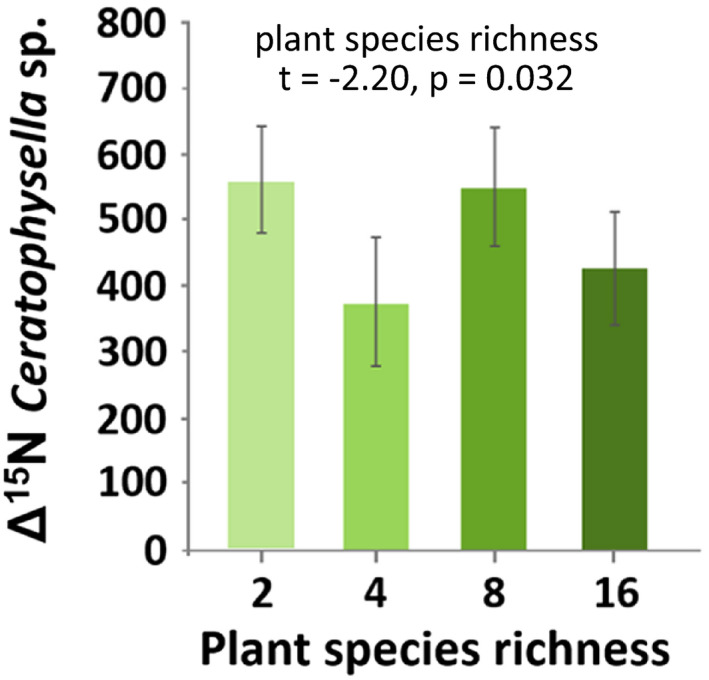
Changes in the incorporation of ^15^N into *Ceratophysella* sp. (Δ^15^N values) with plant species richness; means ± 1 *SE*

**FIGURE 5:**
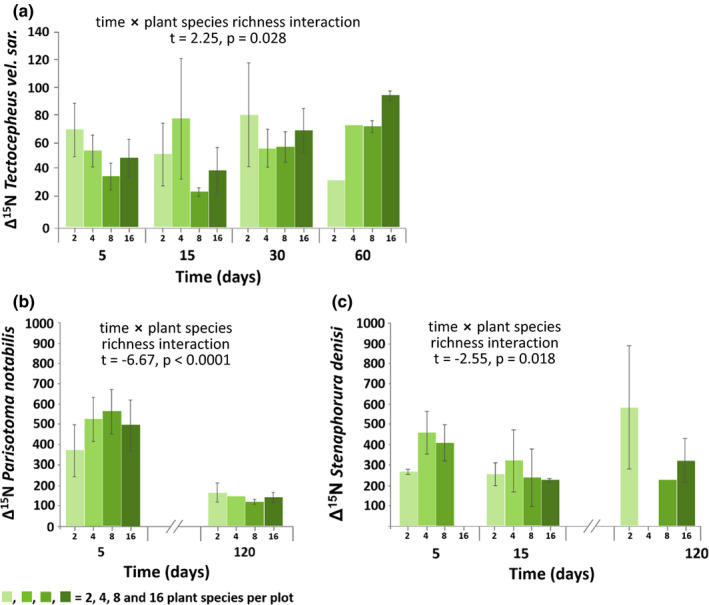
Changes in the incorporation of ^15^N into (a) *Tectocepheus velatus sarekensis*, (b) *Parisotoma notabilis*, and (c) *Stenaphorura denisi* with time and plant species richness (Δ^15^N values); means ± 1 *SE*

**FIGURE 6:**
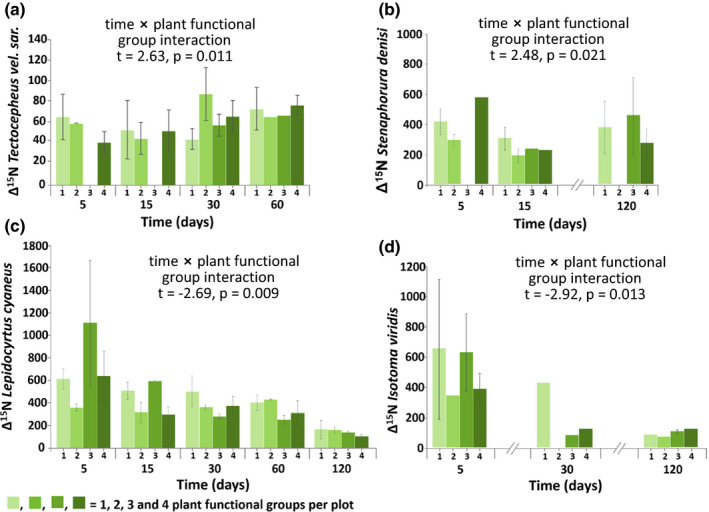
Changes in the incorporation of ^15^N into (a) *Tectocepheus velatus sarekensis*, (b) *Stenaphorura denisi*, (c) *Lepidocyrtus cyaneus*, and (d) *Isotoma viridis* with time plant functional group richness (Δ^15^N values); means ± 1 *SE*

Δ^15^N values of *Ceratophysella* sp. significantly varied with plant species richness being high in plots with two and eight species but low in those with four and 16 species (Figure 4). In *T. velatus sarekensis*, Δ^15^N values also varied with plant species richness, but the effect depended on time. Early in the experiment, the incorporation of ^15^N was lower at high species richness, whereas later it was higher (Figure 5a). Similarly, Δ^15^N values in *P. notabilis* and *S. denisi* also varied significantly with plant species richness and time. In both species, Δ^15^N values increased with plant species richness at day 5, whereas they varied little or decreased at day 120 (Figure 5b,c). Functional group richness as main factor did not affect Δ^15^N values of any studied mesofauna species. However, in combination with time, functional group richness affected Δ^15^N values in *T. velatus sarekensis, S. denisi*, *L. cyaneus*, and *I. viridis* (Figure 6a–d). In *T. velatus sarekensis*, Δ^15^N values decreased with increasing plant functional group richness at day 5, but did not respond in a consistent way at the later sampling dates. In *S. denisi* and *I. viridis,* Δ^15^N values decreased with plant functional group richness at days 15 and 30, respectively, but not at the other days. Similarly, in *L. cyaneus* Δ^15^N values decreased at days 30, 60, and 120, but not at days 5 and 15.

**FIGURE 7:**
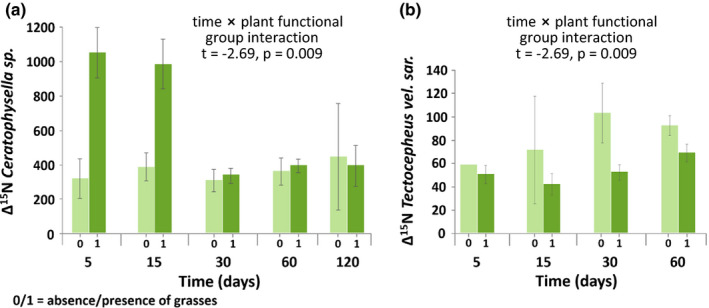
Changes in the incorporation of ^15^N into (a) *Tectocepheus velatus sarekensis* and (b) *Ceratophysella* sp. with time and presence of grasses (Δ^15^N values); means ± 1 *SE*

The presence of legumes generally did not affect Δ^15^N values of any of the studied mesofauna species. By contrast, the presence of grasses significantly affected Δ^15^N values in *Ceratophysella* sp. and *T. velatus sarekensis*, but the effect varied with time (Figure 7a,b). In *Ceratophysella* sp., Δ^15^N values strongly increased in the presence of grasses, in particular at days 5 and 15. By contrast, in *T. velatus sarekensis*, Δ^15^N values decreased in the presence of grasses, with the effect being most pronounced at days 15 and 30.

## 4 DISCUSSION

Soil microorganisms are one of the main food resources of soil animals, thereby channeling microbial N to higher trophic levels of the soil food web. Nevertheless, until today it is unclear which soil animals rely predominantly on N derived from microorganisms and which predominantly rely on N from dead organic matter. Further, it is unknown whether plants modify the uptake of N by soil animals via these channels. Simplified ecosystems with low plant diversity may hamper ecosystem functions (Landis, 2017), including the channeling of N to higher trophic levels. Plant N uptake is driven by plant traits, such as root density and the ability to fix molecular N, and therefore, both plant functional group identity and plant diversity need to be considered for understanding mineral N uptake by microorganisms and its subsequent channeling to higher trophic levels of soil food webs. Despite the need to sustain functioning grassland ecosystems to ensure ecosystem services, detailed knowledge on the relationships between plant community properties and the channeling of N into higher trophic levels of the soil food web is still scarce. To address these gaps of knowledge, we added mineral ^15^N to semi‐natural grassland of different plant diversity and plant community compositions and tracked its incorporation into soil microorganisms and higher trophic levels of the soil food web.

### 4.1 Temporal variation of mineral N incorporation

Generally, the concentration of ^15^N in mesofauna species declined parallel to that in microorganisms with time, reflecting the dominant flux of N from microorganisms to higher trophic levels. The applied ^15^N presumably was increasingly taken up by microorganisms and plants with time. In the long term, plants are superior to microorganisms in mineral N acquisition as they have longer life cycles and effectively capture N released by decaying microorganisms (Hodge et al., 2000; Kaye & Hart, 1997; Kuzyakov & Xu, 2013). In particular in N‐limited ecosystems, such as the grassland site investigated in this study (Strecker et al., 2015), N is immobilized quickly by microorganisms and plants. Mowing and removal of plant aboveground biomass likely aggravate N limitation, even though the deposition of N is high and increasing on a global scale (Leimer et al., 2013, 2016; Reay et al., 2008).

Conform to our expectations (Hypothesis 1), the incorporation of mineral‐derived N into soil animal species followed the incorporation into soil microorganisms. Generally, N incorporation uniformly peaked at the first sampling date after labeling for all studied mesofauna species, except in the primary decomposer *T. velatus sarekensis*. However, the amount of mineral‐derived N incorporated into soil animal tissue differed markedly between animal taxa reflecting differential use of food resources. Further, the incorporation of mineral‐derived N into soil animal species changed with time suggesting that factors driving the incorporation of mineral‐derived N into soil animals, such as tissue turnover and feeding behavior, show species‐specific temporal dynamics.

At the first sampling date, incorporation of mineral‐derived N was highest in the Collembola species *Ceratophysella* sp., followed by the Gamasina *L. berlesei* and the Collembola *L. cyaneus*, *I. viridis*, *P. notabilis, S. denisi*, and the primary decomposer Oribatida *T. velatus sarekensis*. High Δ^15^N values in *Ceratophysella* sp. suggest that this species predominantly acquires its N by feeding on microorganisms, which were heavily labeled with ^15^N. This is supported by other studies reporting *Ceratophysella* sp. and other species of Poduromorpha to predominantly feed on fungi (Chahartaghi et al., 2005; Maraun et al., 2003). However, there is evidence that Poduromorpha species also feed on plants (Sechi et al., 2014); thus, combined feeding on fungi and plant roots may have been responsible for the high Δ^15^N values in *Ceratophysella* sp. Omnivory, that is, feeding on prey from more than one trophic level, may alleviate N limitation by broadening the prey spectrum (Lefcheck et al., 2013; White, 1993). The Collembola species *L. cyaneus* incorporated less ^15^N than *Ceratophysella* sp., but still incorporation was high 5 days after labeling, which also suggests that this species acquired much of its N from feeding on microorganisms. This is in line with other studies reporting *L. cyaneus* to preferentially feed on fungi and to a minor degree on bacteria (Berg et al., 2004; Ferlian et al., 2015). Unexpectedly, also the predatory Gamasina species *L. berlesei* quickly incorporated high amounts of mineral‐derived N similar to the level in its potential prey species *L. cyaneus*. In addition to feeding on highly labeled Collembola prey species, the unexpected fast incorporation of mineral‐derived N into predators may have resulted from feeding on nematodes as major grazers of soil microorganisms (Heidemann et al., 2014). In fact, *L. berlesei* has been shown to prey on nematodes and small arthropods such as Collembola (Christian & Karg, 2006; Walter & Ikonen, 1989), thereby quickly incorporating N from basal resources. Further, *L. berlesei* develops fast, reaching maturity after only 9–19 days (Christian & Karg, 2006), suggesting that this species incorporates mineral‐derived N from prey species within a few days.

Intermediate levels of mineral‐derived N incorporated into animals, such as the Collembola *I. viridis*, *P. notabilis*, and *S. denisi*, suggest that these species only in part fed on microorganisms and that their diet includes a substantial amount of dead organic matter. However, as these species have been assumed to be predominantly microbivorous (Berg et al., 2004; Chahartaghi et al., 2005; Ngosong et al., 2011), slow tissue turnover rate may also have contributed to the lower ^15^N incorporation as compared to the microbivorous *Ceratophysella* sp. and *L. cyaneus*.

Low incorporation of mineral‐derived ^15^N in *T. velatus sarekensis* suggests that this species little relies on microbial N. Notably, only in this species the incorporation of mineral‐derived N increased slowly with time. This suggests that the predominant feeding strategy of *T. velatus sarekensis* was detritivory, potentially including microbial residues, confirming earlier studies assuming this species to live as primary decomposer virtually not relying on N from living microorganisms (Laumann et al., 2007; Maraun et al., 2011; Siepel & Ruiter‐Dijkman, 1993).

### 4.2 Variation of mineral N incorporation with plant diversity

In general, plant diversity played a major role for the incorporation of mineral‐derived N into the studied mesofauna species, and this was true for both plant species richness and plant functional group richness. Confirming our Hypothesis 2, plant species richness decreased the uptake of mineral‐derived N by microorganisms. Competition between microorganisms and plants for mineral N in soil is likely to be aggravated in more diverse plant communities as they take up N more efficiently than communities of low diversity (Bessler et al., 2012; Jesch et al., 2018), for example, due to different rooting depths of different plant species (Cardinale et al., 2007; Scherer‐Lorenzen et al., 2003; Spehn et al., 2005). Despite stronger competition for soil N under high plant diversity, positive effects of plant diversity on microbial communities may surpass negative ones as soil microbial activity and biomass increase with plant diversity, probably due to increased rhizodeposition that mitigates carbon limitation of microorganisms (Cline et al., 2018; Lange et al., 2015; Strecker et al., 2015, 2016).

Also in line with Hypothesis 2, plant species richness as a main factor significantly decreased Δ^15^N values in *C* *eratophysella* sp., likely because *Ceratophysella* sp. fed on microorganisms that were also reduced in ^15^N due to limited N supply in high diverse plant communities (see above). Interestingly, plant species richness exerted time‐dependent effects on *T. velatus sarekensis*, *P. notabilis*, and *S. denisi*. In *T. velatus sarekensis*, Δ^15^N values decreased with plant species richness at the beginning of the experiment, but increased with increasing plant species richness later in the experiment. Potentially, the increase in Δ^15^N values in *T. velatus sarekensis* with plant species richness later in the experiment was due to increased availability of dead plant roots containing ^15^N from the mineral ^15^N added. In *P. notabilis* and *S. denisi*, Δ^15^N values increased with increasing plant species richness early in the experiment, but this effect disappeared later. Presumably, this reflects that certain species of microorganisms associated with roots incorporated more ^15^N in more diverse plant communities and were heavily grazed by microbivorous microarthropods such as *P. notabilis* and *S. denisi*.

In addition to plant species richness, Δ^15^N values of microarthropods also varied significantly with plant functional group richness. In *T. velatus sarekensis*, *S. denisi*, *I. viridis,* and *L. cyaneus,* Δ^15^N values decreased with increasing plant functional group richness in particular early in the experiment. Presumably, this again reflects the more effective capture of nutrients by plants in more diverse plant communities resulting in lower incorporation of ^15^N into microorganisms.

### 4.3 Variations in mineral N incorporation with plant functional group identity

Conform to Hypothesis 3, the presence of legumes decreased mineral‐derived N in soil microorganisms, with the effect being most pronounced at day 30 and declining later in the experiment. This is in line with results of Strecker et al. (2015) reporting that legumes reduce N limitation of soil microorganisms at the field site of the Jena Experiment, presumably via the release of N fixed by legumes into the soil via rhizodeposition diluting the added mineral ^15^N and thereby its uptake by microorganisms. However, we found no effect of legumes on the incorporation of mineral‐derived N into any of the studied mesofauna species. Obviously, the effect of legumes on soil microbial N did not propagate to higher trophic levels, contrasting our expectations. Potentially, the large number of other plant species in the Jena Experiment diluted the legume effect at our field site.

In contrast to Hypothesis 4, the presence of grasses did not affect mineral‐derived N in microorganisms. This contradicts earlier findings showing that the presence of grasses enhances the microbial C‐to‐N ratio, that is, decreases the availability of N for soil microorganisms (Strecker et al., 2015). Although the presence of grasses did not change microbial ^15^N APE, they affected Δ^15^N values in *Ceratophysella* sp. and in *T. velatus sarekensis*. However, the effects differed between species and varied with time. In *Ceratophysella* sp., the presence of grasses increased Δ^15^N values with the effect being strongest at days 5 and 15. The positive effect of grasses on the Δ^15^N values of *Ceratophysella* sp. not only supports the view that the diet of this species is not restricted to microorganisms but also includes plants (Sechi et al., 2014) in particular roots highly labeled with ^15^N (Jesch et al., 2018). Conform to Hypothesis 4, grasses decreased Δ^15^N values in *T. velatus sarekensis*, and this effect was strongest at days 30 and 60. This suggests that *T. velatus sarekensis* mainly fed on herbs and not on grasses. This is supported by the fact that, despite effective nutrient acquisition, grasses have a higher C‐to‐N ratio compared with many legume and nonlegume herbs (Abbas et al., 2013; Bessler et al., 2012), resulting in low food quality.

## 5 CONCLUSIONS

Labeling temperate grassland soil with mineral ^15^N allowed tracking the incorporation of mineral N into soil microorganisms and its transfer into higher trophic levels of the soil food web as affected by plant diversity and community composition. Importantly, the method allowed differentiating between soil animals relying on microbial N and those relying on N from dead organic matter. All of the investigated mesofauna species at least in part incorporated microbial mineral‐derived N. Our data thus underline the predominant role of microorganisms in channeling N to higher trophic levels of soil food webs and indicate that this resource contributes significantly to the nutrition of soil invertebrates. Notably, predatory species quickly incorporated not only mineral‐derived N (within 5 days), suggesting that prey species with short life cycles, presumably mainly nematodes, speed up the channeling of microbial N into predators, but also secondary decomposers such as Collembola, which in part also feed on nematodes.

Confirming our expectations, plant diversity (species richness and functional group richness) significantly modified the incorporation of mineral‐derived N into the studied mesofauna species. The data suggest that high plant diversity reduces the incorporation of mineral‐derived N into higher trophic levels of the soil food web due to their high competitiveness for N toward microorganisms. Thus, we conclude that high plant diversity may alter the competitive interactions between soil animal taxa and change the nutrient transfer across trophic levels in soil food webs via tightening the competition for N. Especially in N‐limited grassland systems such as the field site of the Jena Experiment (Eisenhauer et al., 2010), this may also change the population dynamics and community composition of the soil food web compared to those at low plant diversity. Presumably, high plant diversity may lead to soil food webs that are mainly based on fungi, as these are more efficient in exploiting soil resources compared to bacteria. This is supported by de Vries et al. (2006) and de Vries et al. (2007), who found increased soil fungal biomass in grasslands with no or reduced N fertilizer input (hence, low mineral N availability). In the face of elevated N deposition worldwide, high plant diversity may also buffer detrimental effects of N deposition on soil biota (Eisenhauer et al., 2012), highlighting the importance of sustaining high plant diversity in grassland ecosystems.

## CONFLICT OF INTEREST

The authors declare no competing interests.

## AUTHOR CONTRIBUTIONS

**Tanja Strecker:** Conceptualization (supporting); data curation (lead); formal analysis (lead); investigation (lead); methodology (supporting); software (supporting); visualization (lead); writing – original draft (lead); writing – review and editing (lead). **Annette Jesch:** Formal analysis (supporting); investigation (supporting); visualization (supporting); writing – review and editing (supporting). **Dörte Bachmann:** Formal analysis (supporting); investigation (supporting); visualization (supporting); writing – review and editing (supporting). **Melissa Jüds:** Formal analysis (supporting); investigation (supporting); visualization (supporting); writing – review and editing (supporting). **Kevin Karbstein:** Formal analysis (supporting); software (equal); visualization (supporting); writing – review and editing (supporting). **Janneke Ravenek:** Formal analysis (supporting); investigation (supporting); visualization (supporting); writing – review and editing (supporting). **Christiane Roscher:** Conceptualization (supporting); funding acquisition (supporting); methodology (supporting); project administration (supporting); writing – review and editing (supporting). **Alexandra Weigelt:** Conceptualization (supporting); funding acquisition (supporting); methodology (supporting); project administration (supporting); supervision (supporting); writing – review and editing (supporting). **Nico Eisenhauer:** Conceptualization (supporting); data curation (supporting); formal analysis (supporting); funding acquisition (equal); investigation (supporting); methodology (supporting); project administration (equal); resources (supporting); software (supporting); supervision (supporting); validation (supporting); visualization (supporting); writing – original draft (supporting); writing – review and editing (supporting). **Stefan Scheu:** Conceptualization (lead); data curation (supporting); formal analysis (supporting); funding acquisition (lead); investigation (supporting); methodology (lead); project administration (lead); resources (lead); software (supporting); supervision (lead); validation (lead); visualization (supporting); writing – original draft (supporting); writing – review and editing (lead).

## Data Availability

Data used in this manuscript have been submitted to Pangaea and are preliminary available at https://issues.pangaea.de/browse/PDI‐26860.

## 1

**TABLE A1:** LM table of t‐ and p‐values for the effects of species richness (SR), plant functional group richness (FGR), legumes (LEG), grasses (GR), time, block (1–4), mesofauna species (*Isotoma viridis*, *Lasioseius berlesei*, *Lepidocyrtus cyaneus*, *Ceratophysella* sp., *Parisotoma notabilis*, *Stenaphorura denisi*, *Tectocepheus velatus sarekensis*), and the respective interactions of plant community properties and time on the incorporation of mineral N (Δ^15^N values) of the studied mesofauna species

	Estimate	*SE*	*t* value	*p*‐value
(Intercept)	2.8070	0.07458	37.637	<0.0001***
SR	−0.0351	0.07170	−0.489	0.6252
FGR	−0.0350	0.04000	−0.875	0.3821
LEG	0.0178	0.08193	0.217	0.8281
GR	0.0656	0.07346	0.893	0.3722
Time	−0.0031	0.00100	−3.069	**0.0023****
Block 1	0.0464	0.08214	0.564	0.5730
Block 2	−0.0715	0.04891	−1.461	0.1448
Block 3	−0.0151	0.04730	−0.319	0.7501
Block 4	0.1309	0.04612	2.839	**0.0048****
*Isotoma viridis*	−0.2912	0.07248	−4.017	**0.0001*****
*Lasioseius berlesei*	−0.1475	0.04349	−3.392	**0.0008*****
*Lepidocyrtus cyaneus*	−0.1593	0.04653	−3.425	**0.0007*****
*Ceratophysella* sp.	−0.8506	0.12190	−6.979	**<0.0001*****
*Parisotoma notabilis*	−0.1456	0.05752	−2.531	**0.0118***
*Stenaphorura denisi*	−0.1785	0.05960	−2.995	**0.0003****
*Tectocepheus velatus sarekensis*	−0.9648	0.04739	−20.36	**<0.0001*****
SR × Time	−0.0003	0.00124	−0.232	0.8163
FGR × Time	0.0000	0.00059	0.009	0.9932
LEG × Time	0.0003	0.00117	0.263	0.7925
GR × Time	−0.0002	0.00112	−0.194	0.8465

Note

Significant effects (*p* ≤ 0.05) are given in bold. Asterisks indicate levels of significance (**p* ≤ 0.05, ***p* ≤ 0.01, ****p* ≤ 0.001).
